# Secreted factors from cultured dental pulp stem cells promoted neurite outgrowth of dorsal root ganglion neurons and ameliorated neural functions in streptozotocin‐induced diabetic mice

**DOI:** 10.1111/jdi.13085

**Published:** 2019-06-21

**Authors:** Emiri Miura‐Yura, Shin Tsunekawa, Keiko Naruse, Nobuhisa Nakamura, Mikio Motegi, Hiromi Nakai‐Shimoda, Saeko Asano, Makoto Kato, Yuichiro Yamada, Takako Izumoto‐Akita, Akihito Yamamoto, Tatsuhito Himeno, Masaki Kondo, Yoshiro Kato, Jiro Nakamura, Hideki Kamiya

**Affiliations:** ^1^ Division of Diabetes Department of Internal Medicine Aichi Medical University School of Medicine Nagakute Japan; ^2^ Department of Internal Medicine School of Dentistry Aichi Gakuin University Nagoya Japan; ^3^ Department of Oral and Maxillofacial Surgery Nagoya University Graduate School of Medicine Nagoya Japan; ^4^ Department of Histology and Oral Histology Institute of Biomedical Sciences Tokushima University Graduate School Tokushima Japan

**Keywords:** Conditioned medium, Diabetic polyneuropathy, Stem cells from human exfoliated deciduous teeth

## Abstract

**Aims/Introduction:**

Transplantation of stem cells promotes axonal regeneration and angiogenesis in a paracrine manner. In the present study, we examined whether the secreted factors in conditioned medium of stem cells from human exfoliated deciduous teeth (SHED‐CM) had beneficial effects on diabetic polyneuropathy in mice.

**Materials and Methods:**

Conditioned medium of stem cells from human exfoliated deciduous teeth was collected 48 h after culturing in serum‐free Dulbecco's modified Eagle's medium (DMEM), and separated into four fractions according to molecular weight. Dorsal root ganglion neurons from C57BL/6J mice were cultured with SHED‐CM or DMEM to evaluate the effect on neurite outgrowth. Streptozotocin‐induced diabetic mice were injected with 100 μL of SHED‐CM or DMEM into the unilateral hindlimb muscles twice a week over a period of 4 weeks. Peripheral nerve functions were evaluated by the plantar test, and motor and sensory nerve conduction velocities. Intraepidermal nerve fiber densities, capillary number‐to‐muscle fiber ratio, capillary blood flow and morphometry of sural nerves were also evaluated.

**Results:**

Conditioned medium of stem cells from human exfoliated deciduous teeth significantly promoted neurite outgrowth of dorsal root ganglion neurons compared with DMEM. Among four fractions of SHED‐CM, the only fraction of <6 kDa promoted the neurite outgrowth of dorsal root ganglion neurons. In addition, SHED‐CM significantly prevented decline in sensory nerve conduction velocities compared with DMEM in diabetic mice. Although SHED‐CM did not improve intraepidermal nerve fiber densities or morphometry of sural nerves, SHED‐CM ameliorated the capillary number‐to‐muscle fiber ratio and capillary blood flow.

**Conclusions:**

These results suggested that SHED‐CM might have a therapeutic effect on diabetic polyneuropathy through promoting neurite outgrowth, and the increase in capillaries might contribute to the improvement of neural function.

## Graphical abstract

In this study, we examined whether the secreted factors could be collected from conditioned medium of stem cells from human exfoliated deciduous teeth had beneficial effects on diabetic polyneuropathy. Conditioned medium of stem cells from human exfoliated deciduous teeth might have a therapeutic effect on diabetic polyneuropathy through promoting neurite outgrowth, and the increase in capillaries might contribute to the improvement of neural function.

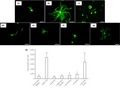

## Introduction

The number of diabetes patients in the world is reported to be >400 million in the IDF DIABETES ATLAS 2017. The purpose of diabetes treatment is to prevent and suppress the progress of diabetic complications that reduce the quality of life and life expectancy of diabetes patients. Diabetic polyneuropathy (DPN) is the most common complication of diabetes mellitus, and affects approximately half of patients with diabetes^1^. The pathological alteration of DPN is degeneration of peripheral nerve fibers and microvascular abnormalities feeding the nerves^2^, ^3^. Existing anti‐DPN drugs merely alleviate the symptoms of pain derived from DPN, but there are no curative remedies effective for pathologically and fundamentally treating DPN^4^.

It has been reported that transplantation of various types of stem cells strengthened nerve regeneration in animal models of several diseases, including spinal cord injury and cerebral infarction^5^, ^6^, ^7^. Recent studies by the present authors and others have also shown that transplantation of various stem cells into limb skeletal muscles improves the reduction of nerve conduction velocities, nerve blood flow, intraepidermal nerve fiber densities and capillary number to muscle fiber ratio in diabetic animal models^8^, ^9^, ^10^, ^11^. However, these studies showed poor differentiation and low survival rates of transplanted cells, suggesting that the improvement of nerve functions and nerve regeneration were attributable to the paracrine effects of transplanted cells, and that the soluble factors secreted from stem cells played a vital role. In addition, it has been reported that many kinds of cells, including Schwann cells and stem cells, secrete exosomes that contain several microribonucleic acids, and show beneficial effects on both the central and peripheral nervous system^12^, ^13^, ^14^. The administration of let‐7i, which is one of the microribonucleic acids, improved DPN in diabetic mice^15^. Therefore, the use of secreted factors, which can be collected as a serum‐free conditioned medium (CM) of stem cells, without the need for cell transplantation and immunosuppressive agents, has recently become a target of scientific research^16^, ^17^.

Stem cells from human exfoliated deciduous teeth (SHED), which reside within the perivascular niche of the dental pulp, express markers for both neural stem cells and mesenchymal stem cells^18^. SHED have a high capacity of proliferation and differentiate into various types of cells, including neural cells, adipocytes, osteoblasts and chondrocytes^18^, and express many genes encoding extracellular and cell‐surface proteins at levels that are at least twofold higher than those in human bone marrow‐derived mesenchymal stem cells^5^, ^19^. Furthermore, SHED are reported to secrete trophic factors that promote axonal regeneration and angiogenesis^20^. These reports have led us to believe that SHED might have greater potential for the treatment of DPN.

In the present study, we aimed to elucidate the effect of SHED‐CM on DPN in streptozotocin (STZ)‐induced diabetic mice.

## Methods

### Preparation of CM from SHED

Exfoliated human deciduous teeth were obtained from 6‐ to 12‐year‐old children and reserved for clinical purposes at Nagoya University Hospital in Nagoya, Japan using approved guidelines set by Nagoya University (H‐73, 2003). SHED were collected from the dental pulp and cultured as previously described^18^. To prepare CM from SHED, SHED were cultured up to 80% confluence in 10‐cm dishes, which contained approximately 6 × 10^6^ cells, washed three times with phosphate‐buffered saline and then cultured in serum‐free Dulbecco's modified Eagle's medium (DMEM) for 48 h at 37°C. The CM was collected and centrifuged for 5 min at 3,000 *g* at 4°C, and the supernatant was collected and used as SHED‐CM, as previously described^21^.

### Extraction of exosome from SHED‐CM

SHED‐CM was subjected to filtration on 0.22‐μm pore filters (Millipore, Billerica, MA, USA), then the CM was ultracentrifuged at 100,000 *g* for 110 min (L‐70; Beckman Coulter, Indianapolis, IN, USA). The precipitate was resuspended with DMEM and used as exosome. The existence of exosome was confirmed by a transmission electronic microscope.

### Separation of SHED‐CM according to molecular weight

SHED‐CM was separated into four fractions (>100 kDa, 20–100 kDa, 6–20 kDa and <6 kDa) according to molecular weight using Amicon ultra centrifugal filter devices with 50,000, 10,000 or 3,000 molecular weight (Millipore).

### Measurement of angiogenic and neurotrophic factors

The concentrations of angiogenic and neurotrophic factors in SHED‐CM were measured with a human enzyme‐linked immunosorbent assay kit (vascular endothelial growth factor [VEGF], basic fibroblast growth factor [FGF2; R&D Systems, Minneapolis, MN, USA], nerve growth factor, brain‐derived neurotrophic factor, neurotrophin‐3, neurotrophin 4/5 [Biosensis Pty Ltd., Adelaide, SA, Australia]), according to the manufacturer's instructions. Absorbance was measured at 450 and 570 nm with a microplate reader (SpectraMax M5; Molecular Devices, Sunnyvale, CA, USA).

### Primary culture of dorsal root ganglion neurons and evaluation of neurite outgrowth

Dorsal root ganglion (DRG) neurons were prepared from 4‐ to 6‐week‐old normal male C57BL/6 mice, as previously described^22^. After anesthesia and taking blood from the hearts, the spines were removed. Using a dissecting microscope, the spinal cord was exposed, and approximately 20 DRGs (Th8–L5) were immediately isolated from each mouse. All the procedures were finished within 30 min per mouse. DRGs were collected into DMEM/F12 (1:1) medium (Gibco; Invitrogen, Carlsbad, CA, USA) and incubated in Leibovitz's L‐15 medium (Gibco; Invitrogen) containing 0.42% collagenase (Wako Pure Chemical Industries, Osaka, Japan) for 30 min at 37°C. After spinning at 230 *g* for 5 min and removing the supernatant, DRGs were dissociated in fetal bovine serum with a flame narrowed glass pipette. After spinning at 230 *g* for 5 min and removing the supernatant again, DRGs were diluted in DMEM or SHED‐CM, which contained 30 nmol/L selenium. Furthermore, either exosome or a fraction of SHED‐CM (>100 kDa, 20–100 kDa, 6–20 kDa or <6 kDa) was added to DRG neurons in DMEM. The isolated DRG neurons were seeded on glass coverslips coated with poly‐L‐lysine in 24‐well plates. Two or three mice were killed per experiment, and DRG neurons were seeded on from six to eight glass coverslips. After culturing for 48 h, DRG neurons were fixed with 4% paraformaldehyde for immunostaining. Neurons were permeabilizated with 0.3% Triton X–phosphate‐buffered saline for 10 min at room temperature and incubated in 1% bovine serum albumin–phosphate‐buffered saline for 30 min for blocking. DRG neurons were stained with Anti‐Neurofilament H Antibody (1:200, MAB5256X; MilliporeSigma, Burlington, MA, USA) for 1 h. This antibody only detects the medium‐to‐large population of DRG neurons. Neurofilament heavy chain belongs to the group of type IV intermediate filaments and has an important function in mature axons. Therefore, we considered that anti‐neurofilament heavy chain antibody was enough to visualize neurons and neurite outgrowth regardless of the population of DRG neurons. For staining the nucleus, coverslips were also stained with 4′,6‐diamidino‐2′‐phenylindole dihydrochloride (Merck, Tokyo, Japan) for 5 min. Images were captured by a charge‐coupled device camera (DP70; Olympus Optical, Tokyo, Japan) using a fluorescence microscope (BX51; Olympus Optical). We observed neurite outgrowth of at least 10 neurons per coverslip four times. All neurites were traced and measured by free‐hand using ImageJ (Research Services Branch of the National Institutes of Health, Bethesda, MD, USA). The average of total neurite length per neuron was analyzed.

### Cell viability of human umbilical vein endothelial cells

The effect of SHED‐CM on viability of human umbilical vein endothelial cells (HUVECs; Lonza, Tokyo, Japan) was examined by 3‐(4,5‐dimethylthiazol‐2‐yl)‐2,5‐diphenyltetrazolium bromide (MTT) assay (Dojindo Laboratories, Kumamoto, Japan). HUVECs were seeded at a density of 1 × 10^4^ cells/100 μL in 96‐well plates. After 24 h, the medium was changed into M199 for serum starvation and HUVECs were incubated overnight. Then, the medium was changed into six different culture media (DMEM, DMEM with 0.5 nmol/L [19.1 ng/mL] VEGF, SHED‐CM, >6 kDa of SHED‐CM, <6 kDa of SHED‐CM and DMEM with exosome). After 24 h, HUVECs were incubated with 100 μL MTT solution (0.5 mg/mL) for 4 h at 37°C. Then 100 μL of 0.04 mol/L HCL/isopropanol was added to dissolve the formed formazan for 10 min at room temperature. The optical density was measured at a wavelength of 570 nm with a microplate reader (SpectraMax M5; Molecular Devices, Sunnyvale, CA, USA).

### *In vivo* study

Five‐week‐old male C57BL/6 mice (Japan SLC, Hamamatsu, Japan) were used for experiments according to a protocol approved by the Department of Animal Experiments in Aichi Medical University. The mice were housed in an aseptic animal room at a temperature of 20–24°C with a 12‐h light cycle and 12 fresh air changes per hour, and allowed free access to food and water. Diabetes was induced by intraperitoneal injection of STZ (150 mg/kg; Sigma‐Aldrich). One week after STZ administration, we checked blood glucose from 09.00 to 12.00 hours under ad libitum feeding, and the mice with plasma glucose concentrations >350 mg/dL were considered to be diabetic mice. Twelve weeks after the induction of diabetes, mice were injected with 100 μL of SHED‐CM or DMEM into their right soleus muscles twice a week over a period of 4 weeks (*n* = 7–10). Before (12 weeks after the induction of diabetes) and after (16 weeks after the induction of diabetes) the injection, weight and blood glucose levels were measured.

### Thermal plantar test

Hind paw withdrawal response against thermal stimuli of radiant heat was measured before and after the treatment in diabetic mice and age‐matched normal mice, using a plantar test 7370 device (Ugo Basile, Comerio, Italy) according to the method Himeno *et al*.^10^ reported. Mice were put on a glass pane and separated into a Perspex enclosure in which the animal is unrestrained. Radiant heat was placed below a glass pane and beamed onto the plantar surface of the hind paw. The paw withdrawal latencies were recorded automatically by a sensor. The latencies were measured six times per session, separated by a minimum interval of 5 min. Paw withdrawals due to locomotion or weight shifting were not counted. Data are expressed as paw withdrawal latency in seconds.

### Measurement of nerve conduction velocities

Mice anesthetized with isoflurane were placed on a heated pad at 37°C in a room maintained at 25°C to ensure a constant rectal temperature of 37°C. Motor nerve conduction velocities (MNCVs) were determined between the sciatic notch and ankle, and sensory nerve conduction velocities (SNCVs) were measured between the knee and ankle, with retrograde stimulation with a Neuropak NEM‐3102 instrument (Nihon‐Koden, Osaka, Japan), as previously described^10^, ^23^.

### Measurement of blood flow in hind paw and sciatic nerve

Hind paw and sciatic nerve blood flow was measured by a laser Doppler Blood Flow Meter (Flo‐N1; Omega Wave Inc., Tokyo, Japan). Mice anesthetized with isoflurane were placed on a heated pad at 37°C in a room maintained at 25°C to ensure a constant rectal temperature of 37°C. A laser probe was placed 1 mm above the plantar skin and the exposed sciatic nerve of mice, as previously reported^10^, ^24^.

### Measurement of intraepidermal nerve fiber densities

Nerve fibers of plantar skin were stained with anti‐PGP 9.5 antibody, as previously reported^25^. Each individual nerve fiber with branching inside the epidermis was counted as one, and nerve fibers with branching in the dermis were counted separately. Six fields from each section were randomly selected for the intraepidermal nerve fiber densities (IENFDs). IENFDs were derived and expressed as epidermal nerve fiber numbers according to the length of the epidermal basement membrane (fibers per mm).

### Capillary number‐to‐muscle fiber ratio

The sections of soleus muscles were cut into 10 μm and used for immunostaining as previously reported, with minor modifications^8^, ^9^. The vascular capillaries were immunostained with rat monoclonal anti‐mouse CD31 (1:20; Dianova, Hamburg, Germany), and visualized with Alexa Fluor 594 goat anti‐rat immunoglobulin G (H+L) antibody (1:200; Abcam, Tokyo, Japan). Images were captured by a charge‐coupled device camera (DP70; Olympus Optical) using a fluorescence microscope (BX51; Olympus Optical). The capillary endothelial cells and the muscle fiber were counted under light microscopy to determine the capillary density. Five fields from each section were randomly selected for the capillary counts. To avoid overestimating the capillary density due to muscle atrophy or underestimating it due to interstitial edema, the capillary density was expressed as the capillary‐to‐muscle fiber ratio.

### Morphometry of sural nerves

Semithin cross‐sections of the proximal sural nerves were cut into 2 μm and stained with toluidine blue. The total complement of sural nerve myelinated fibers was assessed, and the following parameters were obtained: fascicular area, occupancy rate, mean fiber area, mean axonal area, mean myelin area, fiber diameter, axonal diameter, myelin thickness, axonal circularity and G‐ratio. These parameters were obtained by using image processing and ImageJ (Research Services Branch of the National Institutes of Mental Health, Bethesda, MD, USA).

### Statistical analysis

All of the group values were expressed as the mean ± standard deviation. Statistical analyses were made by one‐way anova, with the Bonferroni correction for multiple comparisons.

## Results

### Effect of SHED‐CM on neurite outgrowth of DRG neurons

**Figure 1:**
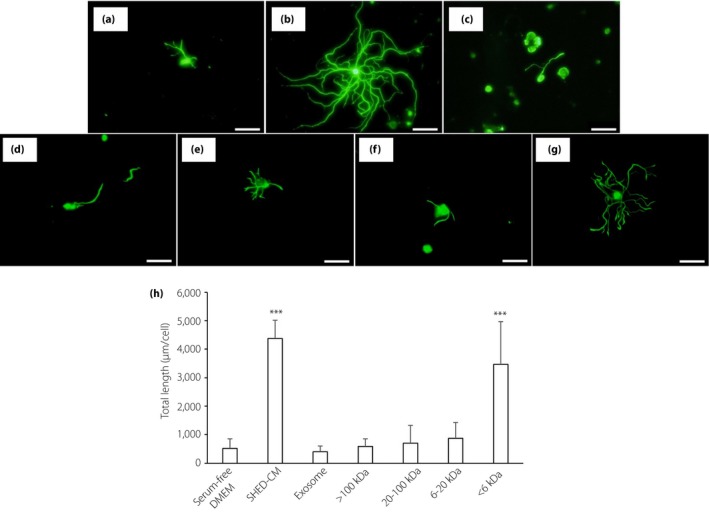
Neurite outgrowth of dorsal root ganglion (DRG) neurons. DRG neurons were cultured with (a) Dulbecco's modified Eagle's medium (DMEM), (b) conditioned medium of stem cells from human exfoliated deciduous teeth (SHED‐CM), (c) exosome, and SHED‐CM of (d) >100 kDa, (e) 20–100 kDa, (f) 6–20 kDa or (g) <6 kDa. Scale bar, 100 μm. (h) Quantitative analyses for neurite outgrowth of DRG. Results are the means ± standard deviation. ***P < 0.001.

We initially examined the effect of SHED‐CM on DPN using a primary culture of DRG neurons from normal mice. DRG neurons were cultured with serum‐free DMEM or SHED‐CM for 48 h. The neurite outgrowth in DRG neurons cultured with SHED‐CM (Figure 1b) were significantly longer than that with DMEM (Figure 1a; SHED‐CM, 4380.3 ± 642.5 μm/neuron; DMEM, 523.4 ± 337.1 μm/neuron, *P *<* *0.001).

### Effects of soluble factors in SHED‐CM on neurite outgrowth of DRG neurons and on the cell viabilities of HUVECs

To evaluate the factors in SHED‐CM that have a beneficial effect on neurons, we extracted exosome from SHED‐CM and separated SHED‐CM into four fractions (<6 kDa, 6–20 kDa, 20–100 kDa and >100 kDa). Incubation with exosome did not improve neurite outgrowth of DRG neurons (401.7 ± 198.7 μm/neuron; Figure 1c), indicating that exosomes purified from SHED‐CM might be not be enough to contribute to the neurite outgrowth of DRG neurons. Among four fractions of SHED‐CM, only the <6 kDa fraction of SHED‐CM promoted the neurite outgrowth of DRG neurons (Figure 1g), and the other three fractions did not show any effect (Figure 1d–f; >100 kDa; 590.4 ± 271.0 μm/neuron, 20–100 kDa; 704.7 ± 619.8 μm/neuron, 6–20 kDa; 873.7 ± 553.4 μm/neuron, <6 kDa; 3467.0 ± 1504.1 μm/neuron, respectively).

We evaluated the concentrations of well‐known neurotrophic or angiogenic factors in SHED‐CM. Whole SHED‐CM contained nerve growth factor, brain‐derived neurotrophic factor, FGF2 and VEGF at concentrations of 9.1 ± 0.3 pg/mL, 69.4 ± 2.4 pg/mL, 7.7 ± 0.8 pg/mL and 1455.9 ± 21.2 pg/mL, respectively. The concentrations of neurotrophin‐3 and neurotrophin 4/5 were too small to detect. As expected, none of these factors, of which the defined molecular weight was >6 kDa, were detected in the fraction of <6 kDa (Figure S1a–d). These results suggested that unidentified soluble factors of <6 kDa in SHED‐CM might have the desired effect on neurite outgrowth.

In MTT assay, SHED‐CM significantly increased the cell viability of HUVECs compared with DMEM. Furthermore, SHED‐CM was more effective compared with DMEM with 0.5 nmol/L (19,100 pg/mL) VEGF (Figure S1e). The concentration of VEGF in SHED‐CM was approximately 1,400 pg/mL, which was very low compared with VEGF added to DMEM. These results suggested that not only VEGF, but also other angiogenic factors in SHED‐CM might have improved the viability of HUVECs.

Next, the effect of SHED‐CM on DPN was investigated in a diabetic animal model. Whole SHED‐CM was administered into the mice, because it contained both the neurotrophic and angiogenic factors in a fraction of >6 kDa and the unidentified factors promoting the neurite outgrowth of DRG in the fraction of <6 kDa, as described above.

### Bodyweight and blood glucose concentrations

Diabetic mice showed severe hyperglycemia and significant reductions in bodyweight compared with non‐diabetic mice, and SHED‐CM administration did not affect plasma glucose levels or bodyweight in non‐diabetic or diabetic mice (Table 1).

**Table 1:** Bodyweight and blood glucose in non‐diabetic and diabetic mice after treatment

	Non‐diabetic mice		Diabetic mice	
	DMEM	SHED	DMEM	SHED
*n*	7	6	10	8
Casual blood glucose (mg/dL)	161 ± 11	168 ± 16	615 ± 276*	607 ± 289*
Bodyweight (g)	27.9 ± 0.4	27.0 ± 2.8	19.4 ± 4.0*	22.7 ± 3.2*

Data are means ± standard deviation.

*P < 0.05 versus non‐diabetic mice.

DMEM, treatment with Dulbecco's modified Eagle's medium; SHED, treatment with conditioned medium of stem cells from human exfoliated deciduous teeth.

### Thermal sensitivities

**Figure 2:**
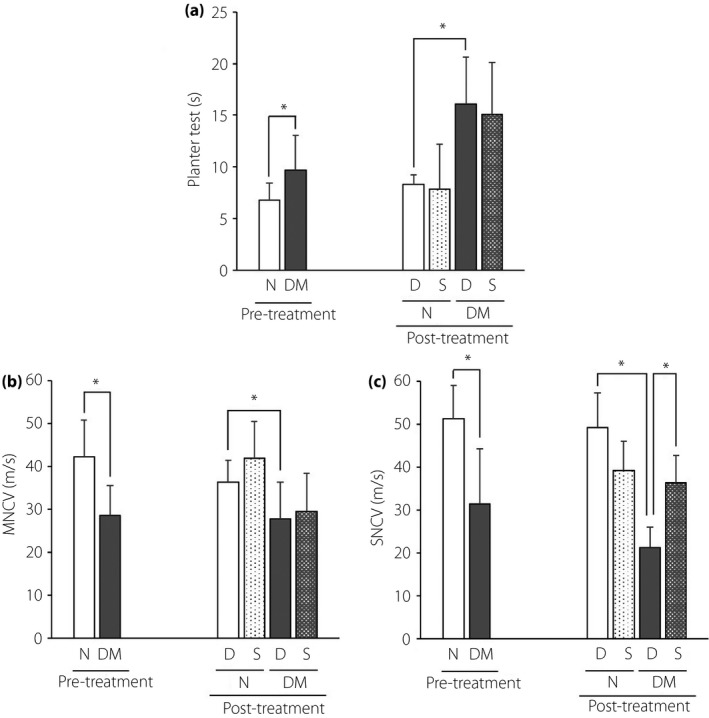
The results of (a) thermal plantar test, (b) motor nerve conduction velocities (MNCV) and (c) sensory nerve conduction velocities (SNCV) were measured before and after the treatment. Results are the mean ± standard deviation. *P < 0.05. D, treatment with Dulbecco's modified Eagle's medium; DM, diabetic mice; N, non‐diabetic mice; Post‐treatment: 16 weeks after streptozotocin injection; Pre‐treatment, after 12 weeks after streptozotocin injection; S, treatment with conditioned medium of stem cells from human exfoliated deciduous teeth.

To evaluate the nociceptive sensitivities, we examined the thermal plantar test. The thermal nociceptive sensitivities became worse in diabetic mice, and SHED‐CM did not prevent the deterioration (Figure 2a). We also used the Von Frey test (data not shown) for evaluating mechanical allodynia. The results tended to become worse in diabetic mice, but did not significantly change by treatment, the same as the thermal plantar test.

### Nerve conduction velocities

MNCVs and SNCVs in diabetic mice were significantly delayed compared with those in non‐diabetic mice. Administration of SHED‐CM significantly prevented the decrease in SNCVs compared with that of DMEM (DMEM, 21.4 ± 4.7 m/s; SHED‐CM, 36.3 ± 6.4 m/s; *P *<* *0.05.). Meanwhile, SHED‐CM showed no effect on the deterioration of MNCVs (Figure 2b,c).

### Morphometry of sural nerves

There were no significant differences in the parameters of morphometric analyses of myelinated fibers in sural nerves between non‐diabetic and diabetic mice. Treatment with SHED‐CM had no effect on the morphometric parameters (Table 2).

**Table 2:** Morphometric data of myelinated fibers in sural nerves

	Fascicle area (μm^2^)	Occupancy rate (%)	Fiber area (μm^2^)	Axon area (μm^2^)	Myelin area (μm^2^)	Fiber diameter (μm)	Axon diameter (μm)	Myelin thickness (μm)	Axonal circularity	G ratio
Non DM‐DMEM	21,111.18 ± 3675.85	50.44 ± 2.59	24.95 ± 1.77	10.04 ± 0.93	15.50 ± 1.38	6.91 ± 0.28	4.72 ± 0.24	1.09 ± 0.07	0.65 ± 0.02	0.67 ± 0.39
Non DM‐SHED	19,589.30 ± 1734.57	55.69 ± 1.74	22.62 ± 2.65	8.25 ± 0.89	13.95 ± 1.81	6.24 ± 0.37	4.19 ± 0.25	1.02 ± 0.09	0.62 ± 0.02	0.68 ± 0.45
DM‐DMEM	22,748.44 ± 1856.93	53.80 ± 2.82	23.18 ± 1.17	8.54 ± 0.71	14.13 ± 0.67	6.68 ± 0.15	4.64 ± 0.13	1.02 ± 0.03	0.58 ± 0.02	0.70 ± 0.22
DM‐SHED	20,863.11 ± 2693.40	55.79 ± 2.95	20.42 ± 1.98	7.54 ± 0.97	12.28 ± 0.99	6.30 ± 0.24	4.26 ± 0.23	1.02 ± 0.04	0.61 ± 0.02	0.68 ± 0.35

Data are the mean ± standard deviation.

DM, diabetic mice; DMEM, treatment with Dulbecco's modified Eagle's medium; non DM, non‐diabetic mice; SHED, treatment with conditioned medium of stem cells from human exfoliated deciduous teeth.

### Intraepidermal nerve fiber densities

**Figure 3:**
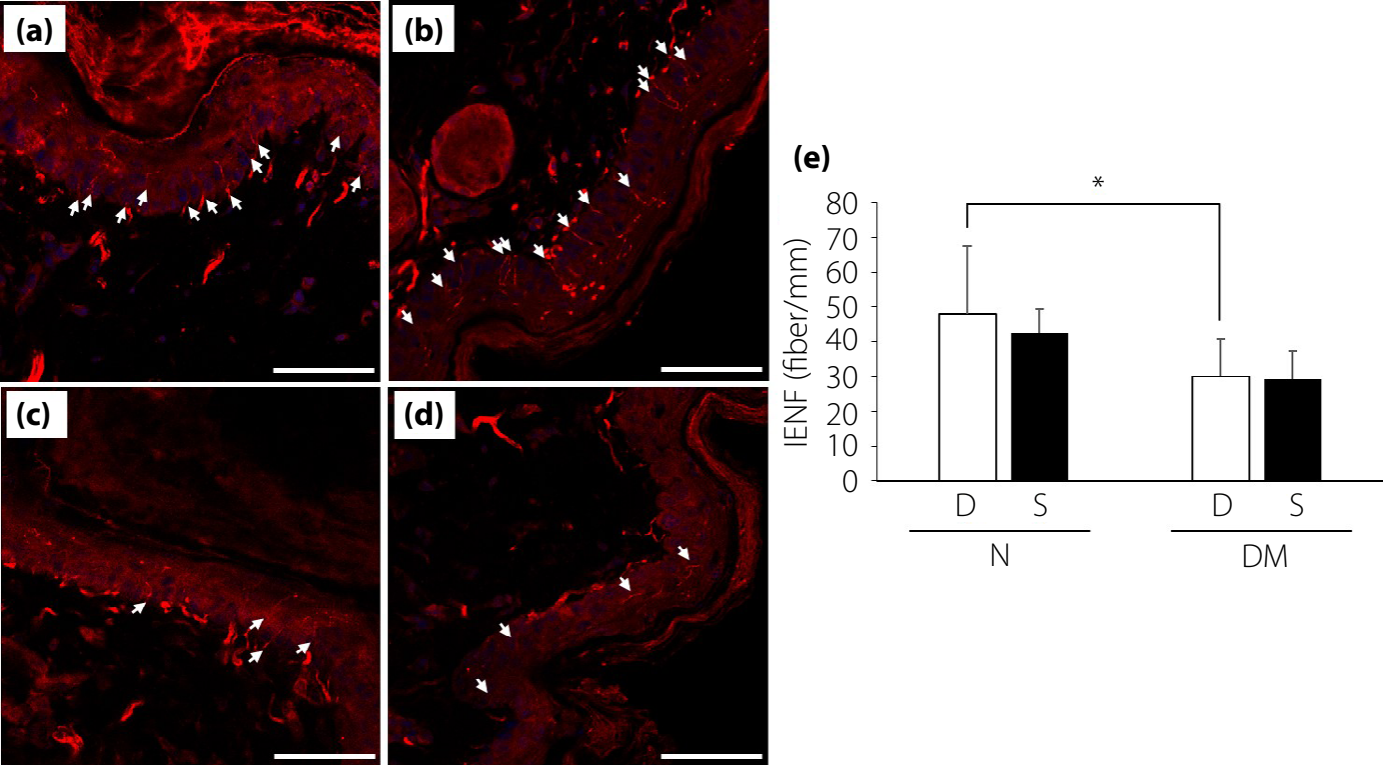
Intraepidermal nerve fiber densities (IENFDs). Non‐diabetic mice treated with (a) Dulbecco's modified Eagle's medium and (b) conditioned medium of stem cells from human exfoliated deciduous teeth, and diabetic mice treated with (c) Dulbecco's modified Eagle's medium and (d) conditioned medium of stem cells from human exfoliated deciduous teeth. Scale bar, 50 μm. White arrows represent intraepidermal nerve fibers. (e) Quantitative analyses for IENFDs. Results are means ± SD. *P < 0.05. D, treatment with Dulbecco's modified Eagle's medium; DM, diabetic mice; N, non‐diabetic mice; S, treatment with conditioned medium of stem cells from human exfoliated deciduous teeth.

Intraepidermal nerve fiber densities were significantly reduced in diabetic mice, and there was no significant difference between treatment with DMEM and SHED‐CM. (Figure 3a–d).

### Capillary number‐to‐muscle fiber ratios, and blood flow in hind paw and sciatic nerve

**Figure 4:**
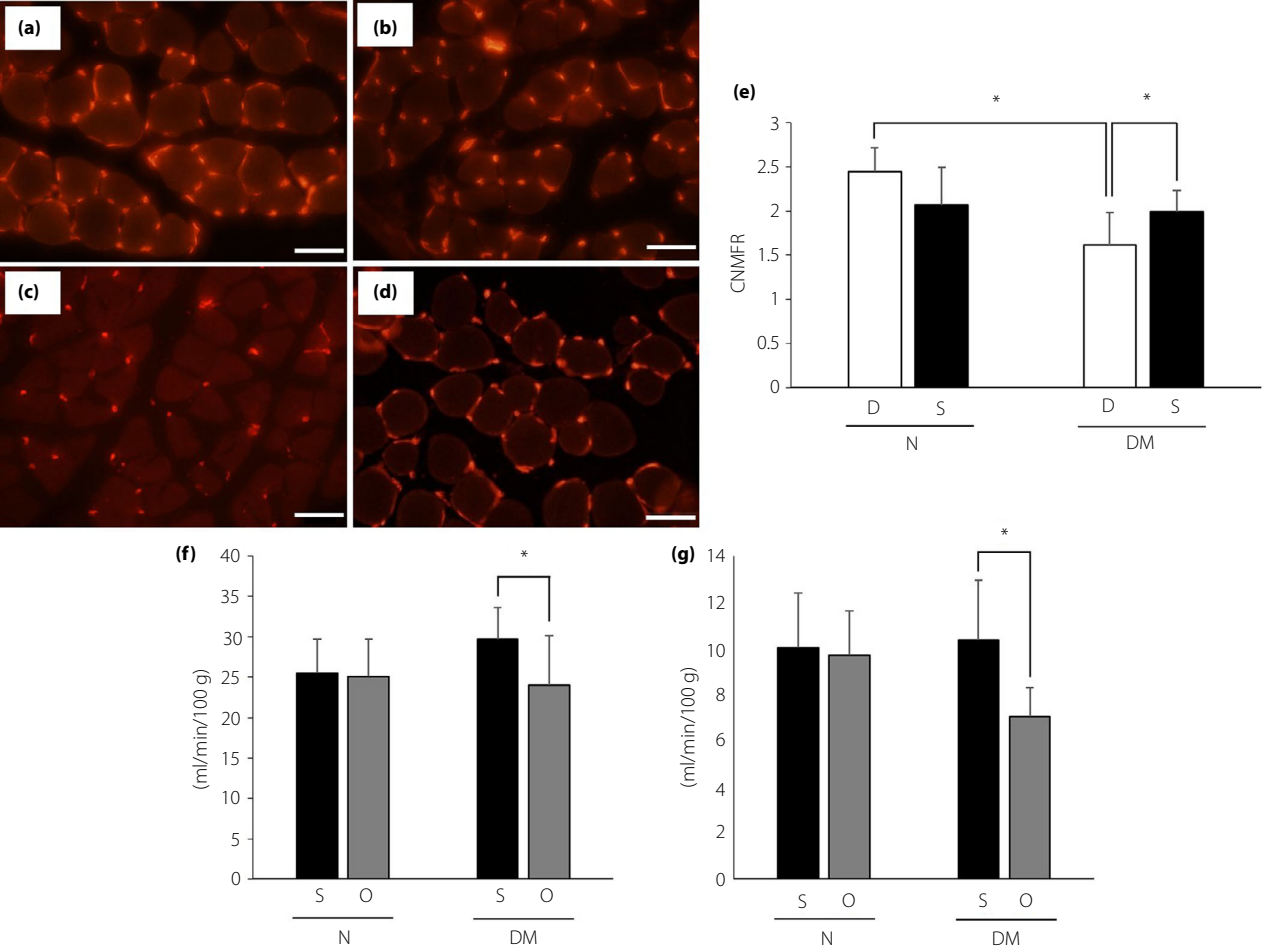
Capillary number‐to‐muscle fiber ratio (CNMFR) and blood flow in hind paw and sciatic nerve. CNMFR of non‐diabetic mice treated with (a) Dulbecco's modified Eagle's medium and (b) conditioned medium of stem cells from human exfoliated deciduous teeth, and diabetic mice treated with (c) Dulbecco's modified Eagle's medium and (d) conditioned medium of stem cells from human exfoliated deciduous teeth were measured after the treatment. Scale bar, 50 μm. Quantitative analyses for CNMFR (e). Blood flow of (f) hind paw and (g) sciatic nerve were also measured after the treatment. Results are the mean ± standard deviation. *P < 0.05. D, treatment with Dulbecco's modified Eagle's medium; DM, diabetic mice; N, non‐diabetic mice; O, opposite side of treatment with conditioned medium of stem cells from human exfoliated deciduous teeth; S, treatment with conditioned medium of stem cells from human exfoliated deciduous teeth.

The capillary number‐to‐muscle fiber ratios in diabetic mice with treatment of DMEM (1.62 ± 0.36; Figure 4c) were significantly decreased compared with those in non‐diabetic mice (2.44 ± 1.62; Figure 4a). The treatment of SHED‐CM increased the ratios in diabetic mice (1.99 ± 0.24, *P *<* *0.05 vs diabetic mice with treatment of DMEM; Figure 4d). The hind paw and sciatic nerve blood flow on the side of treatment with SHED‐CM increased compared with the opposite side in diabetic mice (Figure 4f,g).

## Discussion

In the present study, we showed that SHED‐CM promoted neurite outgrowth of cultured DRG neurons. Intramuscular administration of SHED‐CM alleviated SNCVs in diabetic mice. Instead of no pathohistological findings in the peripheral nerves, capillary numbers in lower limbs were increased in the SHED‐CM‐injected mice. In addition, SHED‐CM increased the viability of HUVECs and blood flow in hind paw and sciatic nerve compared with the opposite side in diabetic mice, indicating that the increase in capillary numbers by SHED‐CM might be associated with an improvement of the sensory nerve function through increasing the nerve blood flow. Furthermore, the present study could be reliable and valuable in terms of clinical use for patients with DPN, because the CM was collected from human stem cells.

Soluble factors from SHED‐CM contributed to the acceleration of neurite outgrowth in the culture system of DRG neurons from normal mice. It has been reported that a variety of cytokines contained in SHED‐CM have a beneficial effect on various diseases^26^, ^27^, ^28^. We previously reported that a fraction consisting of molecules of >100 kDa in SHED‐CM had protective effects on pancreatic β‐cells^21^, suggesting that SHED‐CM might be able to show multiple potent actions for diabetes mellitus. In the present study, the elongation effect of SHED‐CM on the neurite outgrowth was preserved only by the <6 kDa fraction among four fractions with low‐ to high‐molecular weight ranges. It was unlikely that several well‐established neurotrophic factors, such as FGF2, nerve growth factor and NT3, were active molecules for the neurite outgrowth, because these factors were not contained in the small molecule fraction. In addition, the concentrations of such neurotrophic factors in the fractions of >6 kDa were the levels of pg/mL, meanwhile the previously reported effective doses of the factors for neurite outgrowth were the levels of ng/mL^29^, ^30^, ^31^. Furthermore, it is interesting that <6 kDa fraction also increased the cell viability of HUVECs in the present study (Figure S1e). It is still completely unclear what the candidate factor of <6 kDa is in SHED‐CM; therefore, proteomic analysis should be carried out to identify the unknown neuritogenic and angiogenic factors in SHED‐CM in the future. Supplementation of purified exosomes did not promote neurite outgrowth, providing no additional significance to determine the unidentified neuroregenerative molecules. Therefore, our data might predict the existence of undetermined small molecules other than well‐known neurotrophic factors in SHED‐CM for the neurite outgrowth, and further studies to identify the molecules would be required.

We utilized three histopathological approaches to clarify the neuroprotective effects of SHED‐CM in DPN. First, the myelinated fiber morphometry of sural nerves, which evaluated pathological changes in large‐ and medium‐diameter fibers, showed no significant differences even in the comparison between diabetic and non‐diabetic mice. Second, IENFDs that were verified as a standard examination to evaluate small fiber neuropathy in DPN confirmed significant decreases in small fibers in the epidermis of diabetic mice. These findings showed that the duration of diabetes in the current study might be insufficient to induce morphological change in large‐ and medium‐diameter fibers, but enough in small‐diameter fibers. No alteration of thermal and tactile sensitivities in diabetic mice might associate with the result that IENFDs were not improved by SHED‐CM. DPN at 12 weeks after STZ administration progressed to an advanced state of hypoalgesia in the present study, and it would be more effective to start the treatment at an earlier point after STZ administration. Third, the capillary‐to‐muscle fiber ratio of soleus muscles was assessed to evaluate the involvement of microcirculation. The series of SHED‐CM injections increased the ratio in diabetic mice, and increased the sciatic nerve and hind paw blood flow on the treatment side compared with the opposite side in the very same diabetic mice, suggesting that SHED‐CM might ameliorate the microcirculation of lower limbs, including peripheral nerves, resulting in prevention of further deterioration of sensory function.

SHED secreted several angiogenic factors, including VEGF and FGF2, which play a significant role in promoting angiogenesis in tissue regeneration^32^, ^33^, ^34^. The concentration of VEGF in SHED‐CM was relatively higher than those of neurotrophic factors. Therefore, such angiogenic factors in SHED‐CM might be associated with the increase in capillary number‐to‐muscle fiber ratios. The concentration of VEGF in SHED‐CM was approximately 1,400 pg/mL. In the present study, SHED‐CM were injected with 100 μL into diabetic mice twice a week over a period of 4 weeks and the total injected amount of VEGF was approximately 1.2 ng, but a much higher amount was administrated for angiogenesis in *in vivo* studies of previous reports^35^, ^36^. In addition, SHED‐CM improved the cell viability of HUVECs more than the supplementation of 0.5 nmol/L (19.1 ng/mL) VEGF alone. Therefore, not only VEGF, but also various angiogenic factors in SHED‐CM might be associated with the increase in capillary number‐to‐muscle fiber ratios. Meanwhile, it was reported that VEGF induced cancer pain through activation of VEGF receptor expressed in sensory neurons^37^. Thus, we should consider the possibility that VEGF might exacerbate diabetic neuropathic pain in future clinical application of SHED‐CM in DPN.

Previous studies reported that both MNCVs and SNCVs were improved by transplantation of stem cells^23^, ^38^, ^39^, but the present study showed that the deterioration of SNCVs, but not MNCVs, was significantly prevented by SHED‐CM treatment. MNCVs were not further deteriorated during the period of treatment in both DMEM‐ and SHED‐CM‐treated mice in the present study. A decline of MNCVs usually appears subsequently to that of SNCVs in DPN, indicating that deterioration of MNCVs seemed to require longer periods compared with SNCVs due to the difference in nerve fiber size between motor and sensory nerves, and the duration of treatment might be inadequate to evaluate the preventive effect of SHED‐CM on MNCVs. Furthermore, in the previous study, transplanted stem cells were from the same species as the recipient animals, whereas in the present study, CM from human stem cells was administered to mice. The different animal species of the stem cells for CM and the recipient might be associated with the reduced effects of condition medium on improvement of DPN in the present study compared with the previous study. Furthermore, transplanted stem cells were identified at the transplantation site and secreted trophic factors 4 weeks after transplantation^9^, indicating that the transplanted stem cells might have more sustained and pronounced effects on DPN compared with the intramuscular administration of CM. Therefore, it might be important to consider the method of administration of SHED‐CM to effectively induce neuroregeneration in DPN. In rat sciatic nerve transection model, silicon conduits containing SHED‐CM enhanced sciatic nerve reinnervation and regeneration^20^. The continuous intracerebral administration of SHED‐CM significantly improved the neurological outcome in the mice with hypoxic‐ischemic brain^40^, and the continuous intrathecal administration into injured rat spinal cords caused remarkable functional recovery^5^, implying that the effects of SHED‐CM on DPN could be more pronouncedly derived by changing the manner of administration. In addition, a standardized production might be crucial for appropriate utilization of CM, because the concentration of VEGF in the present study was different from that in the previous study^20^, and was changed according to the passage number (data not shown). More suitable methods for preparation and administration of SHED‐CM need to be further elucidated to achieve more remarkable improvement of DPN.

SHED‐CM promoted neurite outgrowths of DRG neurons from normal mice, increased capillaries and prevented the deterioration of SNCVs, suggesting that SHED‐CM might have potential as a new therapeutic strategy for DPN. The use of CM could resolve several problems of cell transplantation: limited survival of transplanted cells, risks of tumor formation or rejection responses. Furthermore, CM is a promising prospect for production as pharmaceuticals for regenerative therapy, because CM could be manufactured, freeze‐dried, packaged and transported more easily. Future studies should be carried out to identify the undetermined small molecules that have favorable effects on nerve regeneration, and to investigate the mechanism of effective actions by SHED‐CM for clinical applications in the treatment of DPN.

## Disclosure

The authors declare no conflict of interest.

## Supporting information


**Figure S1** The concentrations of growth factors, (a) nerve growth factor (NGF), (b) brain‐derived neurotrophic factor (BDNF), (c) fibroblast growth factor (FGF2) and (d) vascular endothelial growth factor (VEGF), in conditioned medium of stem cells from human exfoliated deciduous teeth (SHED‐CM), >6 kDa and <6 kDa of SHED‐CM. (e) The cell viability of human umbilical vein endothelial cells (HUVECS).
