# Supplementary figures and images for: Secreted factors from cultured dental pulp stem cells promoted neurite outgrowth of dorsal root ganglion neurons and ameliorated neural functions in streptozotocin‐induced diabetic mice
Source: J Diabetes Investig. 2019 Jun 21;11(1):28–38. doi: 10.1111/jdi.13085 (PMC6944849; doi:10.1111/jdi.13085)

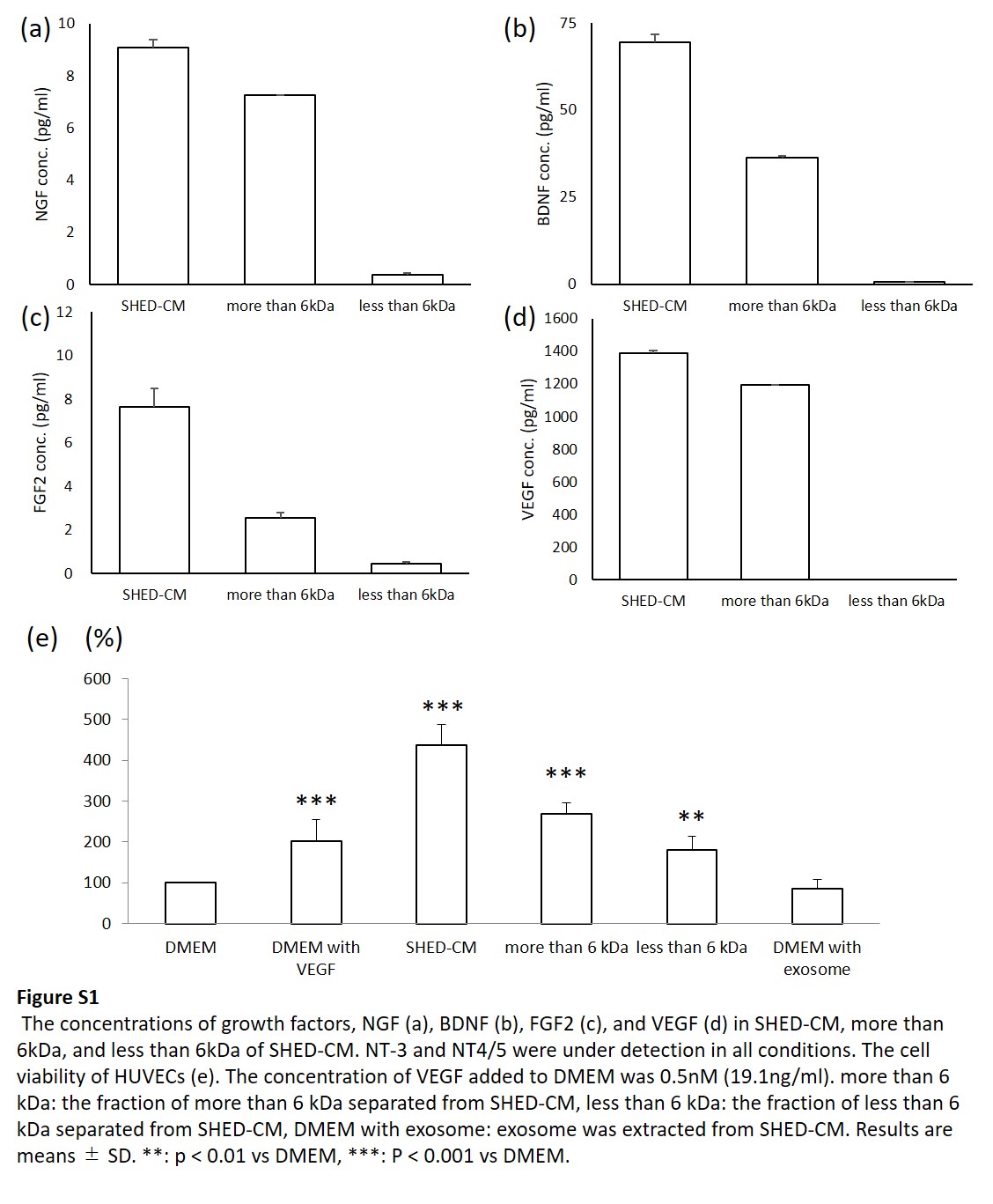

Supplement: Supplementary file 1 — Figure S1 The concentrations of growth factors, (a) nerve growth factor (NGF), (b) brain‐derived neurotrophic factor (BDNF), (c) fibroblast growth factor (FGF2) and (d) vascular endothelial growth factor (VEGF), in conditioned medium of stem cells from human exfoliated deciduous teeth (SHED‐CM), >6 kDa and <6 kDa of SHED‐CM. (e) The cell viability of human umbilical vein endothelial cells (HUVECS). [file JDI-11-28-s001.jpg]
